# Cutaneous Application of Capsaicin Cream Reduces Clinical Signs of Experimental Colitis and Repairs Intestinal Barrier Integrity by Modulating the Gut Microbiota and Tight Junction Proteins

**DOI:** 10.1021/acsptsci.4c00207

**Published:** 2024-06-12

**Authors:** Elandia A. Santos, Janayne L. Silva, Paola C. L. Leocádio, Maria Emilia R. Andrade, Celso M. Queiroz-Junior, Nathan S. S. Oliveira, Juliana L. Alves, Jamil S. Oliveira, Edenil C. Aguilar, Kennedy Boujour, Bruno Cogliati, Valbert N. Cardoso, Simone Odilia A. Fernandes, Ana Maria C. Faria, Jacqueline I. Alvarez-Leite

**Affiliations:** † Departamento de Bioquímica e Imunologia—Instituto de Ciências Biológicas, Universidade Federal de Minas Gerais (UFMG), Belo Horizonte 31270-901, Brazil; ‡ Departamento de Análises Clínicas e Toxicológicas, Faculdade de Farmácia da UFMG, Belo Horizonte 31270-901, Brazil; § Departamento de Morfologia, Instituto de Ciências Biológicas—(UFMG), Belo Horizonte 31270-901, Brazil; ∥ Departamento de Patologia Animal, Universidade de São Paulo (USP), São Paulo 05508-220, Brazil; ⊥ Department of Cellular Biology and Infection, Unity of Biochemistry Membrane and Transport, Institut Pasteur, Paris 75724, France; # Icahn School of Medicine at Mount Sinai, New York, New York 10029, United States

**Keywords:** capsaicin, TRPV cation channels, inflammatory bowel diseases, colitis, ulcerative, dextran sulfate

## Abstract

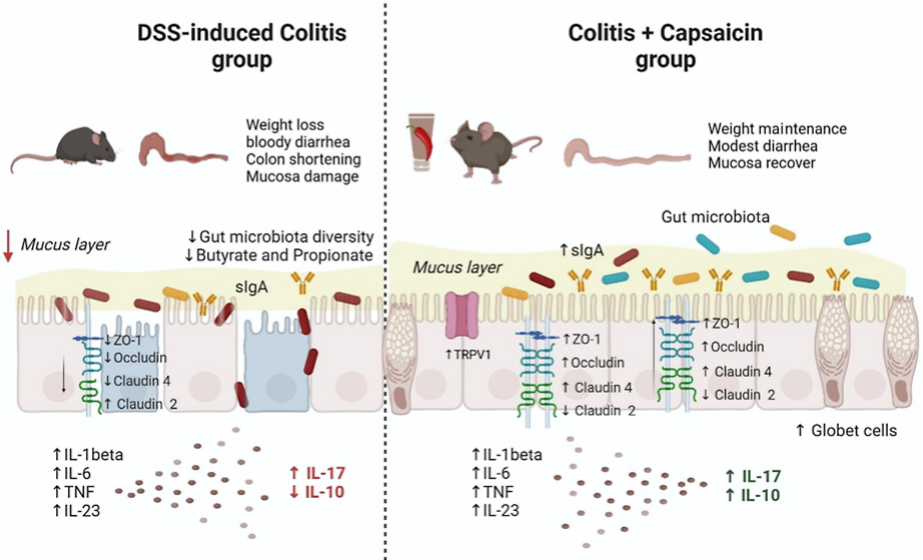

Capsaicin, a pungent compound in chili peppers, is described as having potent anti-inflammatory, antioxidant, and antimicrobial properties. It is also described as a potential modulator of the immune system and intestinal microbiota. Oral or rectal administration of capsaicin has been studied to treat or prevent colitis. However, those vias are often not well accepted due to the burning sensation that capsaicin can cause. Our objective was to evaluate whether the application of capsaicin skin creams (0.075%) would be effective in improving inflammation and epithelial barrier function as well as the composition of the gut microbiota in a model of mild colitis induced by dextran sulfate sodium (1.5%). The results showed that the cutaneous application of capsaicin reversed weight loss and decreased colon shortening and diarrhea, all typical signs of colitis. There was also an improvement in the intestinal epithelial barrier, preserving proteins from tight junctions. We also evaluated the biodistribution of ^99m^technetium-radiolabeled capsaicin (^99m^Tc-CAPS) applied to the back skin of the animals. We found significant concentrations of 99 mTc-Cap in the colon and small intestine after 2 and 4 h of administration. In addition, there was an increased expression of capsaicin receptor TRPV1 in the colon. Moreover, animals with colitis receiving cutaneous capsaicin presented a better short-chain fatty acid profile and increased levels of SIgA, suggesting increased microbiota diversity. In conclusion, our work opens avenues for further studies to better understand capsaicin’s potential benefits and mechanisms in addressing colitis through cutaneous application.

## Introduction

Ulcerative colitis (UC) is a chronic inflammatory disease characterized by relapsing and remitting inflammation of the colonic mucosa. Clinical symptoms include weight loss, abnormal mucus secretion, abdominal discomfort, and bloody diarrhea. Individuals with UC have a shorter life expectancy and are at higher risk for colectomy and colorectal cancer.^1−3^

Capsaicin (8-methyl-*N*-vanillyl-trans-6-nonamide) is a phytochemical derived from plants of the genus *Capsicum*, popularly known as chili peppers. Capsaicinoids have pharmacological properties that could be useful for pain management, weight reduction, cardiovascular protection, cancer prevention, and relief of gastrointestinal diseases. It is considered a valuable nutraceutical agent with therapeutic applications in pain and inflammation control. The main effect of capsaicin is related to interaction^4,5^ with transient potential receptor subtype 1 (TRPV1). The intestines are abundantly innervated by sensory nerves that express TRPV1 channels, and their activation plays an essential role in regulating the function of the microbiota.^6−9^ Capsaicin could also modulate the gut microbiota, influencing its composition and function, increasing the abundance of short-chain fatty acids (SCFAs), especially butyrate, favoring the presence of butyrogenic bacteria.^10−13^

Due to its pungency, the use of capsaicin orally or rectally has been associated with discomfort and burning in the gastrointestinal tract, especially in the anal region, which induces patients with inflammatory bowel diseases to avoid chili peppers’ intake. Due to its chemical structure, capsaicin is well absorbed by the skin, and creams and lotions are already used to treat neuropathic pain in several countries. However, few studies have been directed to the effect of cutaneous application of capsaicin on intestinal inflammation under conditions such as colitis.

The dextran sulfate sodium (DSS) model of colitis is commonly used to study UC. It causes intestinal inflammation similar to that of human UC. DSS is a toxic agent to the colonic epithelium, resulting in epithelial cell injury. The efficacy and intensity of DSS-induced colitis depend on various factors.^14^ Fang et al.^54^ identified 1609 genes significantly altered during DSS colitis, related to inflammation, angiogenesis, metabolism, and other responses. Compared with UC patient data, 152 genes were similarly upregulated and 22 were downregulated. Temporal genome-wide expression profile analysis of DSS-induced colitis revealed associations with immune responses and tissue remodeling events similar to those in UC patients.

We aimed to evaluate if cutaneous application of capsaicin could reach the colon and reduce clinical manifestations, intestinal barrier alterations, and dysbiosis seen in an experimental DSS-induced colitis model.

## Results

### Capsaicin and Vehicle Cream Purities

The analysis of both creams’ purity performed by high-performance liquid chromatography (HPLC) is shown in the Supporting Information (Figure S1).

### Capsaicin Reduces Clinical Manifestations of DSS-Induced Colitis

**Figure 1:**
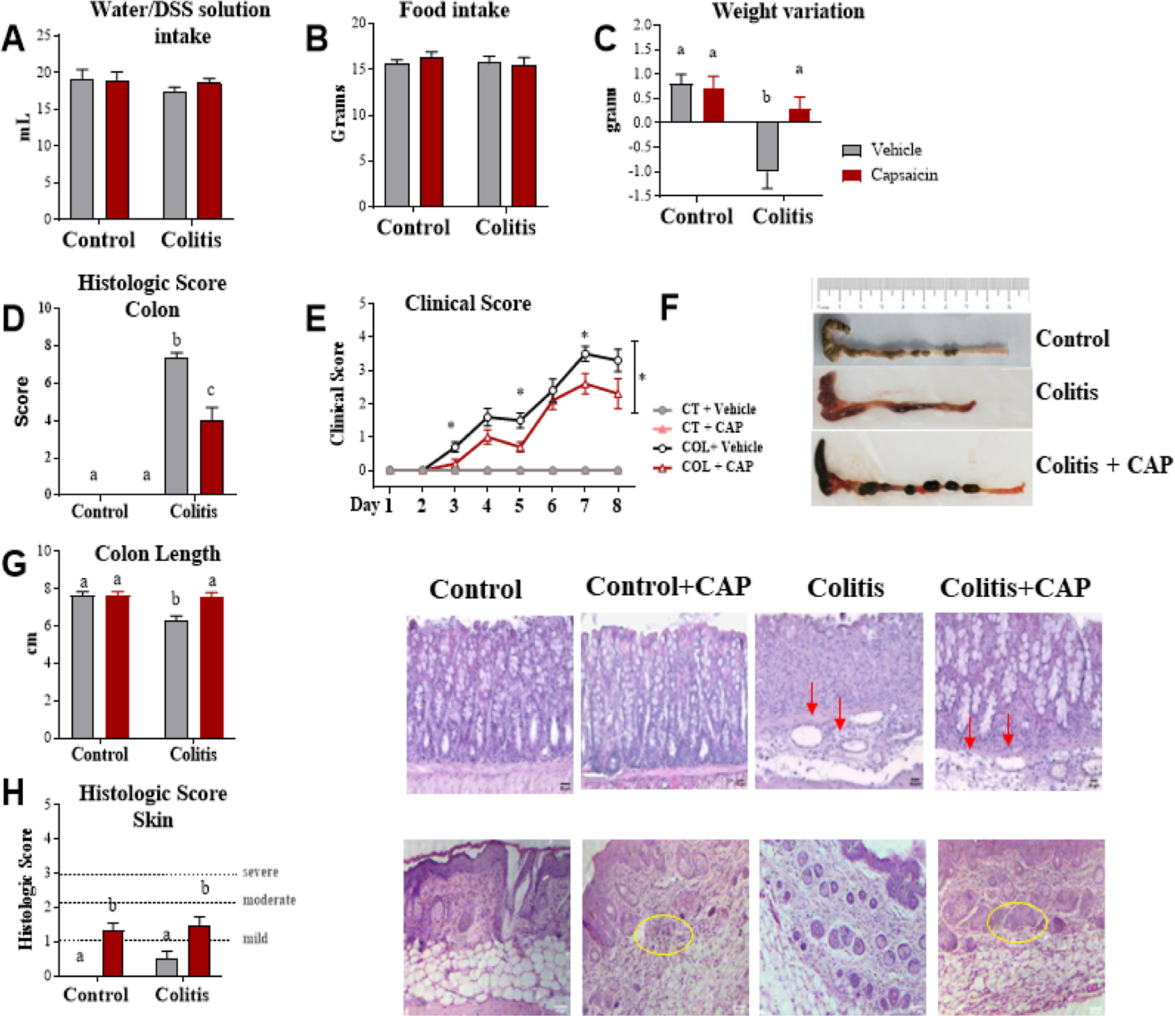
Total water/DSS solution (A) and food (B) intake; body weight variation (C), clinical score evolution (D), colon length (E,F), and the histopathological score of the colon (G) and skin (of control or DSS-induced colitis mice treated with topical cream of capsaicin (0.075%) or vehicle for 5 days). The bars represent the mean, and the vertical lines represent the standard error. Scale bar: 50 nm. Different letters mean statistical difference (*p* < 0.05). Two-way ANOVA test. In (G), the yellow arrowhead = goblet cells; red arrows = inflammatory infiltration; in (H), yellow circles = inflammatory focus. *N* = 10 mice/group except for histopathological scores and colon length (*n* = 6).

During the experiment, the intakes of liquids (water or DSS solution) and diet were similar between groups (Figure 1A,B). Despite similar intake, animals from the colitis group showed a more intense weight loss (Figure 1C) and diarrhea with bloody stools indicated by the clinical score (Figure 1D). Compared to the colitis group, animals from the colitis + capsaicin group showed less intense signs of colitis, with no weight loss, lower intensity of diarrhea, and fecal blood. Colon shortening, an important feature of DSS-induced colitis, was seen in colitis but not in colitis + capsaicin animals (Figure 1E,F). The histopathologic analysis revealed moderate to severe lesions in the colon of the colitis group. The lesions were characterized by areas with loss of tissue architecture and significant disorder of the mucosal structure, with reduction in the number of goblet cells, moderate inflammatory infiltrate, and edema. It is possible to identify that the colitis + capsaicin group showed a reduction in those alterations and histopathological scores (Figure 1G). When we analyzed the skin at the application site, it was possible to see a mild to moderate degree of injury only in both groups receiving capsaicin (Figure 1H).

### Capsaicin Reverses Damage to the Intestinal Epithelial Barrier

**Figure 2:**
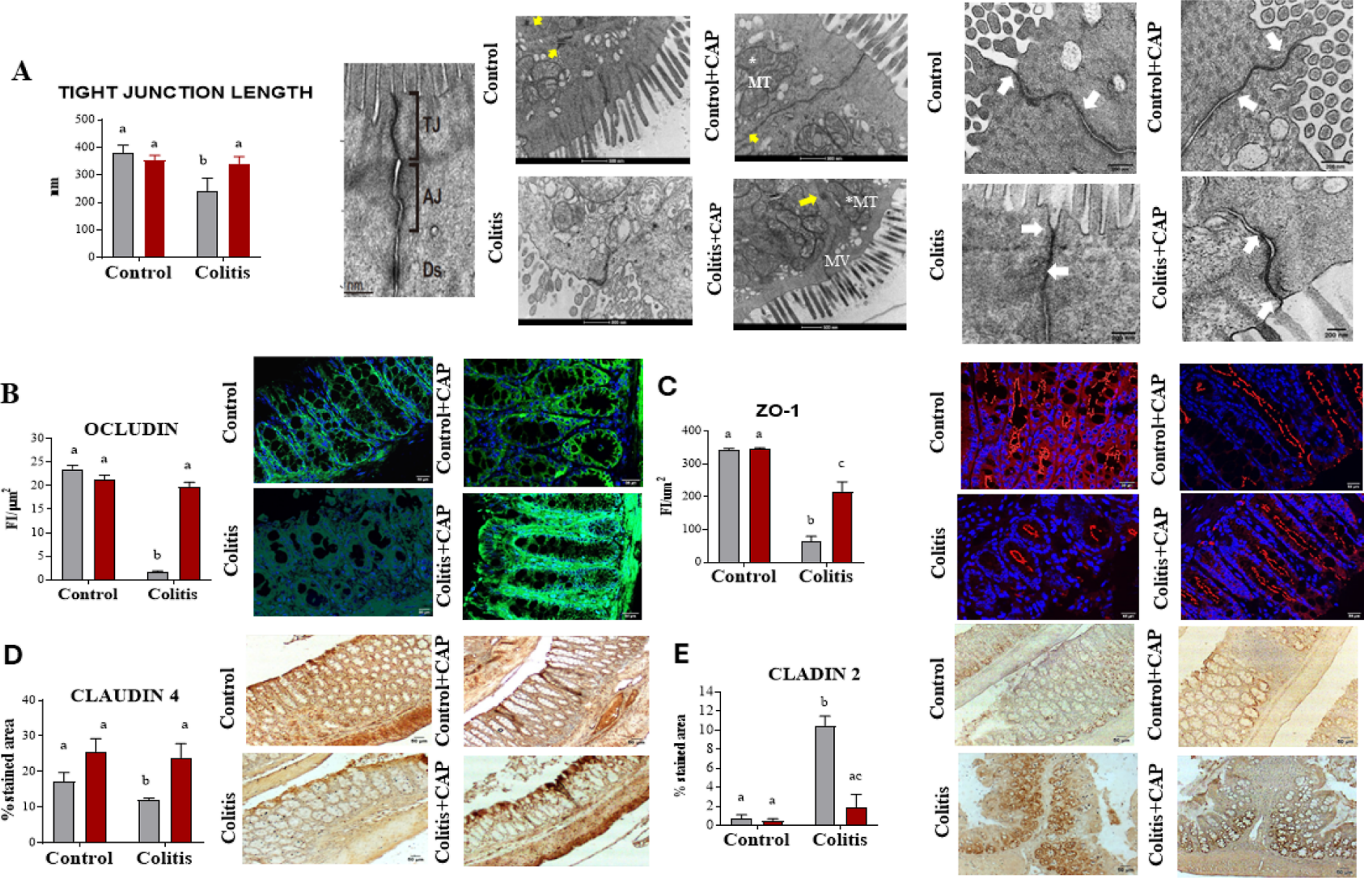
Junctional complex analysis of the colon of control or DSS-induced colitis mice treated with topical cream of capsaicin (0.075%) or vehicle for 5 days. (A) Electronic microscopy and quantitative analyses were performed on 95 junctions in 274 randomly selected electron micrographs, confirming the reduction of the junctional complex. Scale bar: 500 nm. Immunofluorescence analysis of the T protein occludin (B, green) and ZO-1 (C, red). (D,E) Immunohistochemistry of claudin-2 and -4, respectively. Scale bar: 50 nm. Bars represent media and vertical lines represent the standard error. Different letters mean statistical difference (two-way ANOVA). MT = mitochondria, MV = microvilli, AF = actin filaments, yellow arrow = desmosomes, green arrows = length of TJ, and *n* = 5 mice/group.

Next, we studied the junctional complex of the colon to evaluate the effects of the cutaneous application of capsaicin on the integrity of the intestinal barrier (Figure 2). The transmission electronic microscopy confirmed the pattern of mucosa architecture disorganization seen in the histology of the colitis group (Figure 2A) and evidenced a tight junction (TJ) shortening in this group. In the colitis + capsaicin group, cell architecture and the length of the TJ are like both control groups.

We also quantified TJ proteins that could be involved in the changes seen by electron microscopy. The results showed a reduction in the levels of occludin (Figure 2B), ZO-1 (Figure 2C), and claudin-4 (Figure 2D) and an increase in claudin-2 (Figure 2E) protein expression in the colitis group. These findings were associated with the displacement of ZO-1 to the basal region of the cell and the absence of claudin-4 staining in the basolateral regions of the colitis group. However, in the colitis + capsaicin group, the expression and localization of these proteins were partially or totally reestablished.

### Capsaicin Increases the Concentration of IL-17 and IL-10 in the Colon

**Figure 3:**
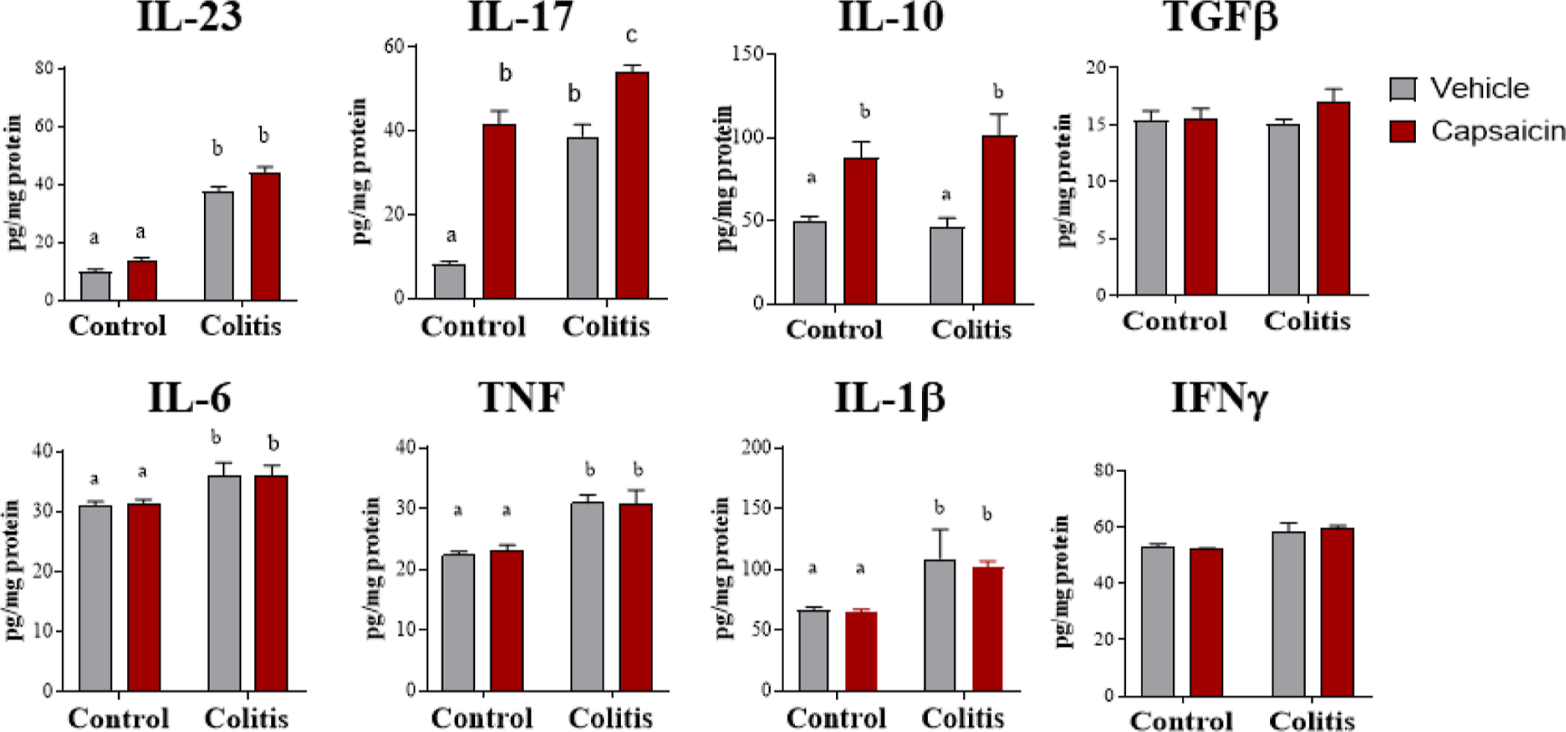
Cytokine levels on the colon of control or DSS-induced colitis mice treated with a topical cream of capsaicin (0.075%) or vehicle for 5 days. Bars represent media and vertical lines represent the standard error. Different letters mean statistical difference (two-way ANOVA). *N* = 5 mice/group.

Cytokine concentration was determined to assess the colitis intensity and the effect of capsaicin cream on these immune parameters. Concentrations of the cytokines IL-23, IL-17, IL-6, TNF, and IL-1β were higher in the colon from the colitis group compared to the control groups, confirming the presence of colitis (Figure 3). However, IL-10, IFN-γ, and TGF-β were not affected by the moderate colitis induced in our experiments. Moreover, cutaneous application of capsaicin increased colonic IL-17 and IL-10 levels regardless of colitis induction. No other effect of capsaicin was evidenced in the other cytokines.

Based on the improvement of colitis with topical capsaicin treatment, our next step was to evaluate whether this effect was due to the presence of capsaicin at the site of injury (colon) or indirectly by a modulation of the immune response in peripheral lymph nodes that could be reflected in the colon inflammation. For this, a cream containing capsaicin radiolabeled with ^99m^technetium (^99m^Tc-CAPS) 3.7 MBq was applied on the last experimental day, and its biodistribution was evaluated 2 and 4 h after application.

The stability of ^99m^Tc-CAPS staining was previously tested, as shown in Supporting Information (S3).

**Figure 4:**
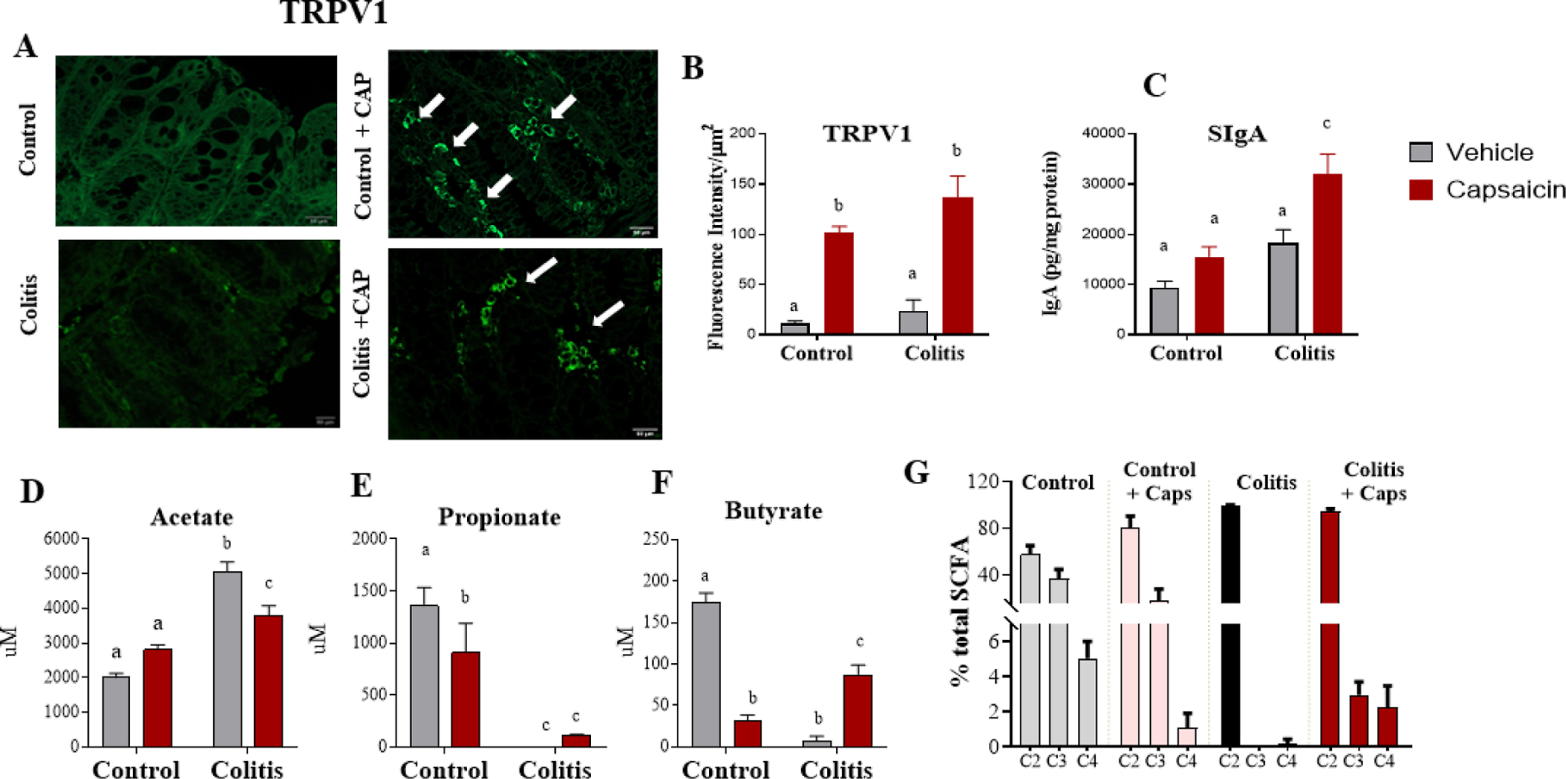
Biodistribution of ^99m^Tc-CAPS cream. (A) Scintigraphic image of the skin of the control (superior images) and colitis (inferior images) of the skin from local cream application. (B) Mice in a supine position, showing the main sites of ^99m^Tc-CAPS deposition and absence of thyroid radiation accumulation. (C) Scintigraphic images of the small intestine and colon of control + caps and colitis + caps groups. (D) Organ biodistribution after 2 and 4 h of cream application. Bars represent media and vertical lines represent the standard error. *Statistical difference from other organs. ^#^Statistical difference from other organs except the lung, colon, and kidney (two-way ANOVA). *N* = 8 mice/group.

The stability of radiolabeled capsaicin was confirmed by the absence of radioactivity in the thyroid region (Figure 4A). If the binding of technetium atoms with capsaicin presented instability, the free technetium (^99m^TcO_4_) would be uptake by the thyroid due to its similar characteristics to the iodine ion.^16^

The results of ex vivo studies showed that ^99m^Tc-CAPS was concentrated mainly in the small intestine and colon of both groups 2 and 4 h after administration (Figure 4D). The biodistribution data are confirmed by the scintigraphic images of the small intestine and colon obtained after euthanasia, where the high concentration of capsaicin in these organs is observed (Figure 4B,C).

**Figure 5:**
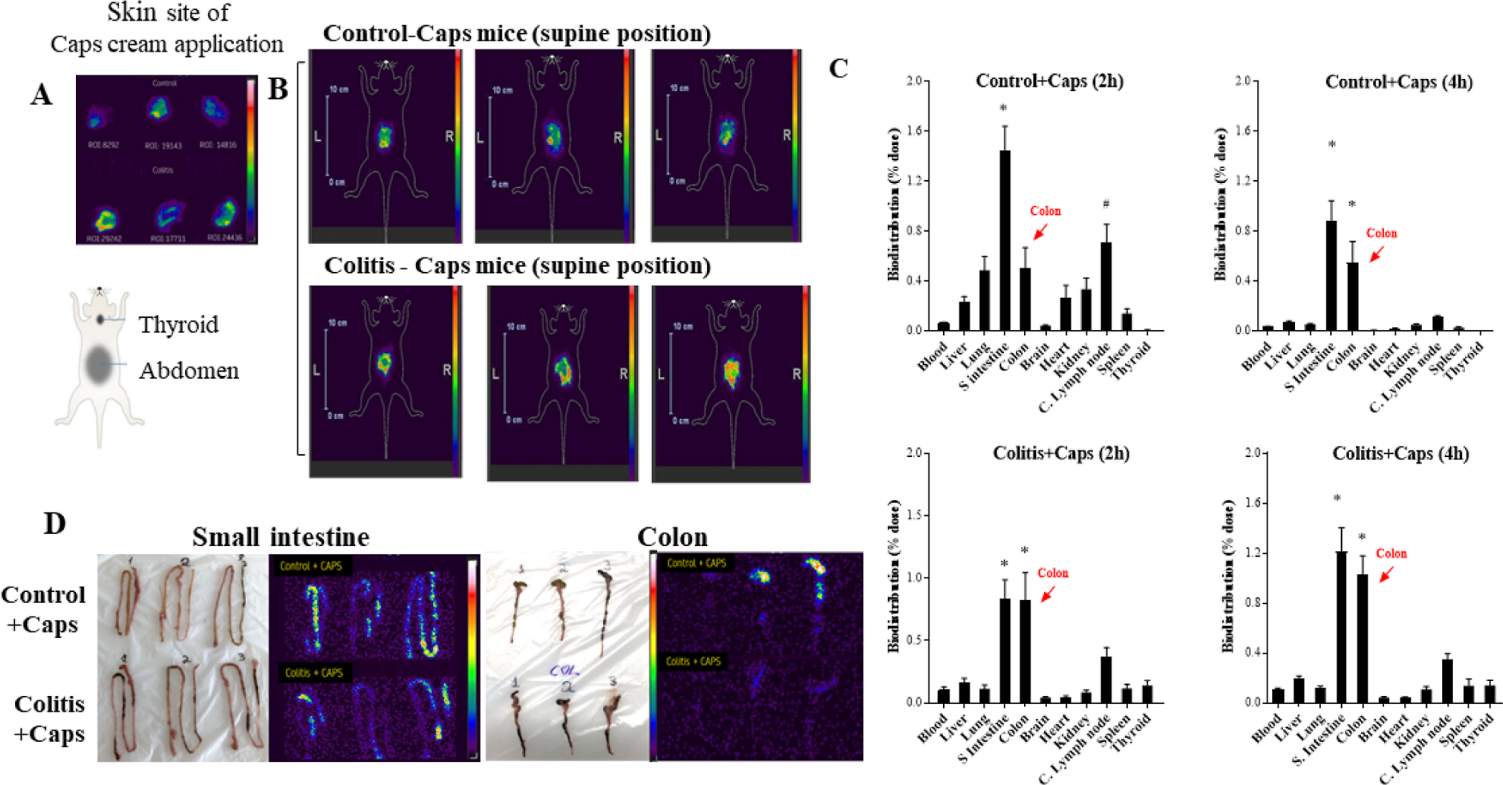
Effect of topical capsaicin application on the expression of TRPV1 receptors in the colon (A,B), secretory IgA in the stool (C), and SCFAs in the cecum homogenate (D,F) of control animals or those with DSS-induced colitis. *N* = 5 mice per group.

To confirm the direct effect of capsaicin on the colon, we also quantified TRPV1 receptors in this organ (Figure 5A,B). Mice from both capsaicin groups had a higher expression of this receptor in the colon, regardless of the presence of colitis.

### Capsaicin May Have a Beneficial Effect on the Maintenance of the Gut Microbiota

First, we measured the concentration of secretory immunoglobulin A (SIgA), which is related to healthy microbiota. The results show an increase in SIgA levels only in animals from the colitis + capsaicin group (Figure 5). Next, we measured the concentration of the SCFA acetate, propionate, and butyrate, which are produced by bacterial fermentation. These fatty acid concentrations are altered by changes in the intestinal microbiota. It was observed that colitis causes an important change in the concentration and proportion of SCFA. In the colitis group, there was an increase in acetate and a drastic reduction in propionate and butyrate concentrations (Figure 5D–F). Interestingly, control + capsaicin mice showed a decrease in propionate and primarily in butyrate levels compared to the control group, indicating that capsaicin cream could impact intestinal microbiota. However, when animals with colitis were given capsaicin, there was a reduction in acetate and an increase in butyrate concentration, showing a profile more similar to those observed in the control group (without capsaicin).

## Discussion

DSS-induced colitis is characterized by a reduction of mucus-producing goblet cells, migration of inflammatory cells to the colonic lamina propria and submucosa, and mucosal ulceration (in high concentration). This study showed that capsaicin applied cutaneously reaches the colon and improves the clinical manifestations (weight loss, intestinal bleeding, shortening of the colon) and the histological signs of mild colitis. It is particularly interesting since capsaicin creams in similar concentrations are already commercially available in many countries. In addition, phytochemicals are a potential beneficial adjuvant in the treatment of inflammatory diseases because they have fewer and less frequent side effects when compared to the drugs available for the treatment of UC.^17−20^

The pharmacological activity of capsaicin depends on factors such as the dose, route of administration, and its concentration in target tissues.^4,21^ In the present study, we chose to use dermatological creams rather than oral intake due to their pungency, which hinders the ingestion of high concentrations. Furthermore, oral capsaicin presents a metabolism different from that when absorbed by the skin or after rectal application. When administered orally, about 50 to 90% of capsaicin is rapidly absorbed from the stomach and intestine by a passive process, rapidly reaching the liver, where it undergoes metabolization.^5^ On the other hand, when applied to the skin, capsaicin is also rapidly absorbed and follows first-order kinetics through the skin barrier. Thus, capsaicin applied to the skin reaches the systemic circulation without metabolization by P450 enzymes in the liver, as occurs in oral administration.^5,22^ In this way, the cutaneous application of capsaicin could reach the target organ, such as the intestine, before hepatic metabolization. In an unprecedented way, using radiomarkers, we evaluated the biodistribution of technetium-labeled capsaicin in several tissues and observed that 2 and 4 h after topical application, a large part of the capsaicin is found in the intestines. These data are corroborated by the increase in TRPV1 receptors in the colon of animals receiving topical capsaicin, indicating the direct action of capsaicin in the colon.

The gut immune system is always challenged with bacterial and food antigens. Goblet cells are responsible for producing a mucus layer that acts as a physical barrier separating the antigens present in the intestinal lumen from the immune system^23^ This mucus layer is composed predominantly of mucin glycoprotein MUC2, along with SIgA. In our colitis model, we found a lower presence of mucus-producing goblet cells that was recovered by capsaicin application, which was associated with the increased SIgA, suggesting an improvement of barrier integrity.

UC results from an interaction of multiple risk factors, such as genetics, gut microbiota, immune system, and environment.^24,25^ Regarding the cytokine profile, cytokines classically considered to have a pro-inflammatory profile, such as IL-1β, IL-6, TNF, IL-17, and IL-23, were increased in the colitis group as expected and demonstrated in previous studies with the colitis model.^26^ The application of capsaicin was responsible for the increase in IL-10, a cytokine with anti-inflammatory characteristics, regardless of the colitis induction.^27,28^

Interestingly, capsaicin also increased the IL-17 concentration in the colon of both control and colitis mice. IL-17-producing Th17 cells develop in the gut in response to commensal microbiota, particularly segmented filamentous bacteria (SFB). Mice lacking SFB in their microbiota have weaker immune responses and are more vulnerable to infections like *Citrobacter rodentium* due to weakened intestinal barrier function.^29^ Moreover, a clinical trial administering a neutralizing IL-17A monoclonal antibody to patients with IBD did not provide protection and was linked to increased adverse events, including *Candida albicans* infection. This suggests that enteric IL-17 responses may be beneficial in the gut.^30^ As seen in our SCFA results and described in the literature, capsaicin is able to change microbiota.^31^ We hypothesized that the increase in IL-17 levels in the control + capsaicin group could be due to the capsaicin’s potential effect of changing the microbiota, possibly increasing SBF in control mice. However, more studies are needed to confirm this hypothesis.

These data seem to be conflicting but should be analyzed in the context of our other findings in our model of mild colitis. It is known that IL-17 expression is significantly increased in patients with active UC. It has also been shown in murine models of colitis that IL-17 produced by Th17 cells and/or by innate lymphoid cells and stimulated by IL-1β and IL-23 play a critical role in chronic intestinal inflammation.^32^ However, IL-17 may act beneficially by promoting the production of antimicrobial peptides and increasing epithelial barrier function to prevent the spread of pathogens.^32^ Thus, we believe that the increase in IL-17 caused by capsaicin has a more protective profile, especially when analyzed considering the improvement in intestinal permeability, TJ proteins, SIgA, and IL-10 concentrations. Nonetheless, in a study of DSS-induced colitis in rats, treatment for 4 weeks with oral capsaicin 12 mg/kg reduced increased levels of IL-17A and INF-γ, which was not in agreement with our study.^33^ In addition to the differences between animal models, capsaicin administration route, and study extent, colitis induced was more severe in the study by Lian et al.,^33^ since the animals received DSS at a concentration of 5% in contrast to our milder colitis induced with 1.5% DSS.

It is not well understood whether IBD-related mucosal permeability is a primary event or a consequence of local inflammation. In the case of Crohn’s disease (CD), Turpin et al. (2020) showed in their prospective study that intestinal hyperpermeability may precede the onset of the disease, indicating that abnormal intestinal permeability and disorganized intestinal barrier increase susceptibility to the development of CD.^34^ Repair of the intestinal epithelium increased the expression of firm junctions, as seen in our study, allowing the reduction of the intestinal inflammatory response.^2,35^ In our model of mild colitis, the more significant effect of capsaicin was reducing the intestinal barrier disruption and improving the microbiota profile, as suggested by SCFA determination. These effects were more significant than minimizing inflammatory cytokines since the increase in IL-10 and IL17 levels in capsaicin groups occurred regardless of colitis induction, and the other tested cytokines, although increased in the colitis group, were not affected by capsaicin treatment. However, other inflammatory markers not assessed in our study could be modified by capsaicin application.

A TJ is a protein complex established by interactions between proteins such as claudin and zonula occludens families. Besides their effect on barrier integrity, occludin and ZO-1 have essential noncanonical (nonbarrier) functions that allow them to regulate apoptosis and epithelial proliferation and organize specialized epithelial structures.^36,37^ ZO-1 is a cytosolic protein with multiple domains that are specialized for protein interactions. These domains allow ZO-1 to bind to several other firm junction proteins, including claudin, F-actin, occludin, ZO-2, and ZO-3.^36^ Our findings show that ZO-1 is reduced in the colitis group and has migrated to the basal region of the cell, corroborating the study by Poritz (2007).^38^

Occludin participates in the maintenance of the barrier between cells on the apical and lateral sides. We found a lower expression of occluding in our colitis group. This reduction was like those seen in the intestines of individuals with UC, Crohn’s disease,^39^ and irritable bowel syndrome.^40^ These changes in ZO-1 and occlusion were partially reversed by the application of capsaicin. The same was seen by Kumar et al. (2022), who kept mice receiving a high-fat diet and capsaicin by gavage for 12 weeks and observed that the reduction in ZO-1 and occludin induced by the high-fat diet was reversed in the group receiving capsaicin^41^

Changes in claudin expression result in outcomes, such as changes in the immune response barrier dysfunction with increased permeability in cases of DSS-induced colitis. The protein expression of claudin-4 is decreased in the intestine of patients with UC,^42^ like what was found in our experimental model. In the colons of healthy individuals, these proteins are found in the basolateral and apical regions of the epithelium. Our results show not only a decreased expression in the colitis group but also the absence of staining in the basolateral regions, an effect not observed in animals treated with capsaicin, where this expression is re-established. These effects appear to be a consequence of the improvement of colitis induced by topical application of capsaicin. Claudin-2 protein expression is consistently increased in the gut of patients with Crohn’s disease and UC,^43^ also in agreement with our findings. Claudin-2 forms channels that regulate TJ’s permeability to water.^44^ The expression of this protein is increased by inflammatory cytokines, which results in an increase in its level in colitis mice. This increase in claudin-2 expression in colitis acts as a protective mechanism against diarrhea by inducing selective cation channels of TJ, increasing the paracellular permeability of Na^2+^ and water. Compared to the colitis group, the animals treated with capsaicin had lower expression of this protein, corroborating our data of a lower incidence of diarrhea in the colitis + capsaicin group.^44,45^

UC has an important component of dysbiosis involved in its etiology and activity. Maintaining microbiota homeostasis is a prospect in the treatment of UC, and restoring microbiota balance may alleviate colitis in mice.^46^ The gut microbiota composition in healthy patients differs from that of individuals with gut inflammation. Zhu et al. (2022) analyzed the gut microbiota in UC patients and healthy individuals and found that the gut microbiota was significantly less abundant and diverse in UC patients than in healthy control subjects. Those with UC also had higher levels of potential pathogens and lower levels of butyrate-producing bacteria.^47^ The mechanisms by which capsaicin modulates gut microbiota have not been completely elucidated in the literature. It has been described that diets enriched with capsaicin and its derivatives increase the abundance of intestinal bacteria, facilitating colonization by *Faecalibacterium prausnitzii* and *Roseburia*, which are important butyrate-producing bacteria necessary for the control of energy metabolism and for the balance of microbiota.^19^ In addition, capsaicin has been associated with a decreased abundance of LPS-producing Gram-negative bacteria and inhibition of the growth of pathogenic bacteria due to a bactericidal effect.

Although we did not analyze gut microbiota, our results showed that DSS-induced colitis mice presented an important reduction in butyrate and propionate concentrations, which suggested a change in microbiota diversity. The reduction in acetate production and increased butyrate production in the animals of the colitis + capsaicin group indicate an SCFA profile consistent with the increase in diversity, especially due to the increase in butyrogenic bacteria, as already described in previous studies.

SIgA is also an important protective component that influences the gut microbiota. A significant fraction of the gut bacteria is coated with SIgA, which recognizes pathogenic bacteria, viruses, and fungi, directing their elimination and preventing intestinal translocation and disease.^48^ SIgA plays a crucial role in the mucosal surfaces lining the gastrointestinal tract. SIgA is involved in the maintenance of gut homeostasis by neutralizing pathogenic microorganisms and toxins, downregulating inflammatory responses, regulating the composition of the gut microbiota, and protecting against inappropriate immune responses to antigens from microorganisms and foods.^49,50^ Our results show increased SIgA in animals treated with capsaicin, reinforcing the set of results suggesting that the topical application of capsaicin can modulate the intestinal microbiota, contributing to the resolution of colitis.

The role of TRPV1 has been repeatedly investigated in animal models of colitis, and despite several studies, its role in protecting or inducing intestinal inflammation is still controversial.^51^ It can be attributed to the different phenotypes and phases of IBD, the involvement of TRPVs in cell signaling pathways, the immune environment, and the limitations of experimental approaches.^51^ Although some studies show TRPV1 activation in UC, in our experimental model, this increase was not statistically significant compared with the control mice. Only groups with cutaneous application of capsaicin showed an increase in the concentration of this channel, indicating that the modulatory effects of capsaicin are at least partially dependent on TRPV1. Based on the other data, we believe that this receptor played an important role in the regulation of intestinal function, modifying inflammatory and immunological conditions in the intestinal environment, as previously described.^52^ In addition, TRPV1 is essential for mucin production and for the preservation of a healthy bacterial population.^19,52,53^

Our study has some limitations. We only investigated mild-severity, acute experimental colitis, and did not explore the long-term application of topical capsaicin in a model of chronic colitis. Therefore, we cannot state that the results would be similarly beneficial in the case of chronic colitis. Additionally, we did not fully explore the role of capsaicin in microbiota, which is an important area for future research.

We hypothesize that when applied topically, capsaicin is absorbed and reaches the colon, where it exerts its main effects. This idea is supported by histological results, the kinetics of labeled capsaicin, and the increased presence of TRPV1 receptors in the colon of colitis + capsaicin mice. The presence of capsaicin in the colon reduces inflammation, as indicated by lower levels of NAG, MPO, and certain inflammatory cytokines, thereby reducing DSS-induced inflammation. Additionally, it improves the microbiota profile, as shown by an improved SCFA profile and higher IgA levels. These combined effects strengthen the integrity of the gut barrier, preventing the translocation of bacteria and antigens and consequently reducing the clinical signs of colitis, such as weight loss, colon shortening, diarrhea, and alteration of colonic architecture.

## Conclusions

Our results show, for the first time, that the cutaneous application of capsaicin cream ameliorates the clinical and histological signs of DSS-induced mild colitis. This effect was associated with a reorganization of TJ proteins, higher secretion of SIgA, and an improvement in the SCFA profile by mechanisms that involve a greater expression of TRPV1. Our results open avenues for further studies on the use of capsaicin creams as an adjunct in the treatment of mild-intensity colitis.

## Methods

### Animals and Experimental Design

C57BL/6 female mice aged between 8 and 10 weeks were acquired at the Central Vivarium of UFMG and kept in a standard environment (22 ± 2 °C and 12 h day/night cycle) with free access to food and water (or DSS solution). Experiments were carried out strictly following the guidelines of the Animal Research Ethics Committee, which were approved by the Ethics Committee on the Use of Animals of UFMG – CEUA/UFMG, by protocol no. 399/2017. The animals were divided into the control (vehicle and capsaicin) and colitis (vehicle and capsaicin) groups. The control groups were provided with unlimited access to water, whereas the colitis groups were given unrestricted access to a low concentration (1.5%) of DSS solution instead of water to induce mild colitis. Our goal was to induce colitis without intense ulcerations and inflammation to better observe the presence of capsaicin effects. The mice were shaved on the back (1 cm^2^) for application of the vehicle or 0.075% capsaicin cream (100 mg) on the skin from the third to the last day of DSS administration. After the colitis induction, we started the capsaicin application to observe its effect on treating rather than preventing colitis. The composition of the creams is shown in Table S1.

For colitis induction, 1.5% DSS (w/v) (DSS, 36–50 kDa, MP Biomedicals, no. 160110) was dissolved in water and offered to the colitis groups for 7 days, according to the description of Wirtz et al. (2017).^14^ Throughout the experimental period, animals from all groups were given paracetamol (1 mg^–1^ mL) to reduce pain and allow feeding, minimizing the effects of low dietary intake on colitis. After 2 days of DSS administration, the application of 100 mg of capsaicin cream (0.075%) or vehicle was initiated in a region of 1 cm^2^ of the back of mice near the nape, preventing the animal’s access to the creams. The animals were kept in individual cages to avoid cross-contamination and received the respective creams and solutions until the seventh experimental day. After that, all mice were euthanized for blood and organ collection.

### Clinical Evaluation

Body weight, stool consistency, and fecal bleeding were recorded daily to assess the induction and severity of the colitis. Weight loss was determined by the difference between the final and initial weight, measured on the first and last experimental days. The clinical score considered stool consistency and the presence of fecal blood, assessed visually and by detection cards (INLAB Diagnóstica, Brazil), respectively. The total clinical score was obtained by the sum of the two parameters according to Wirtz (2007).^15^ Stool consistency score: 0 = normal; 1 = soft but still formed; 2 = very soft; and 3 = diarrhea. Fecal blood score: 0 = negative for occult blood, 1 = positive for occult blood, 3 = traces of blood visible in the stool, and 4 = rectal blood. At the end of the experiment, the mice were anesthetized with ketamine–xylazine and euthanized by cervical dislocation. The colon was isolated and its length was measured. Colonic tissue at 1 cm from the anus was excised, rinsed with saline solution, and stored in buffered formalin (10%) for histopathological analysis. The histopathological score considered the presence and extent, on a scale of 0 (absence) to 3 (severe damage), of the following alterations: destruction of mucosal architecture; cell infiltration, ulceration, and crypt size reduction. The histopathological score of the skin at the site of cream application considered the general alterations on a scale from 0 (no lesions) to 3 (severe damage). The colon portion not used for histology was opened longitudinally, and the feces were washed with phosphate-buffered saline (PBS) and stored in an ultrarefrigerator for further analysis.

### Capsaicin Biodistribution

Capsaicin biodistribution was performed by radiolabeling capsaicin with ^99m^technetium (^99m^Tc), using synthetic capsaicin (*N*-vanyllnonanamide—Sigma V9130) and ^99m^Tc in the form of sodium pertechnetate (Na^99m^TcO_4_), obtained from the ^99^molybdenum/^99m^technetium generator (IPEN/CNEN, São Paulo, Brazil). The radiolabeling of the phytochemical and determination of the radiolabeling yield were based on the methodology proposed by Hosseinimehr et al., 2010.^16^ Radiolabeling yield was determined using the formula: % ^99m^Tc-CAPS = 100 – (% ^99m^TcO_4_^–^ + % ^99m^TcO_2_).

After 7 days of treatment, the biodistribution of ^99m^Tc-CAPS was evaluated in control + capsaicin and colitis + capsaicin. The animals were fasted for 12 h, and then 100 mg of cream containing 3.7 MBq (100 μCi) of ^99m^Tc-CAPS was applied as usual. At 2 and 4 h after the cream administration, the animals were anesthetized for blood collection and then euthanized by cervical dislocation for organ harvesting, following the same protocol as in the experimental design. Dose standards containing the same amount of radioactivity administered to the animals were used to correct the physical decay of ^99m^Tc and to calculate the percentage (% dose/g) in each organ investigated. The results were expressed as a percentage of the administered dose per gram of tissue (% dose/g), calculated by the following equation:  (cpm = count per minute).

### Scintigraphic Images

After the ^99m^Tc-CAPS prepared in the cream form 14.8 MBq (400 μCi) was administered on the skin of previously anesthetized mice, scintigraphic images of each animal were obtained after 2 and 4 h (*n* = 3). During scintigraphic imaging, the animals were anesthetized and placed in the prone position on a gamma-camara for small animals equipped with a low-energy collimator (Nuclide TH22, Mediso, Hungary). Images were acquired using a 256 × 256 × 16 pixels matrix size with a ±10% energy window set at 140 keV for 10 min.

### Histopathological Evaluation

The mice’s colonic tissue was embedded in paraffin and sectioned after fixation in paraformaldehyde for 24 h. Hematoxylin–eosin (HE) staining was used for histological analysis. All histological images were captured under an optical microscope. Inflammation was scored in a masked manner using the scoring system described by MacPherson and Pfeiffer, adapted by Costa, 2016. The results were presented as the sum of the scores obtained for each parameter.^17^

### Analysis of the Junctional Complex by Transmission Electron Microscopy

The colonic tissue was collected and processed according to a previous study.^18^ Briefly, tissues were fixed in a mixture of freshly prepared aldehydes [final concentration of 1% paraformaldehyde and 2.5% glutaraldehyde (EM grade, 50% aqueous, Electron Microscopy Sciences-EMS, Hatfield, PA)] in 0.1 M sodium phosphate buffer, pH 7.4, for 2 h at room temperature (RT). All samples were post-fixed in 2% aqueous osmium tetroxide and 1.5% potassium ferrocyanide in 0.1 M sodium phosphate buffer, pH 6.0 (reduced osmium) before dehydration and embedding as above. After polymerization at 60 °C for 16 h, thin sections were cut using a diamond knife on an ultramicrotome (Leica, Bannockburn, IL). Sections were mounted on uncoated 200-mesh copper grids (Ted Pella) before staining with lead citrate and electron micrographs were obtained by a transmission electron microscope (Tecnai G2-20-ThermoFischer Scientific/FEI 2006, Eindhoven, The Netherlands) at 120 kV in the Center for Microscopy of UFMG. All electron micrographs were analyzed in a masked manner to describe the cell-to-cell interaction to investigate morphological changes in the TJ. A total of 95 TJs were counted and their widths were assessed. Quantitative studies were performed using the Image-J (NIH).

### TJ Protein Study

For immunofluorescence staining, nonspecific ligands of the colonic tissue were blocked with 1% serum bovine albumin solution (BSA, Sigma-Aldrich #A7030) followed by application of the primary antibody anti-ZO-1(SC33725 Santa Cruz Biotechnology) and antioccludin (SC8145 Santa Cruz Biotechnology) overnight at 4 °C. After the sections were washed with blocking solution, the secondary antibodies, antigoat-IgG FITC (SC2024 Santa Cruz Biotechnology) and antigoat-IgG Alexa Fluor 594 (R37119 Thermo Fisher Scientific) (1:200 dilution in 1% BSA solution and 0.05% saponin), were applied and used to detect the markings of occludin and ZO-1, respectively. Afterward the recommended time, the sections were washed with a blocking solution and assembled with a mounting medium containing DAPI. The images were captured in an ApoTome.2 ZEISS microscope, and the fluorescence intensity was analyzed with the aid of the image analyzer software Image-J (NIH).

To evaluate the distribution of claudin-2 and claudin-4 proteins, histological sections (5 μm) of the colonic tissue of the animals were previously fixed in 10% formaldehyde, perfused in paraffin, and then submitted to immunohistochemistry. The histological sections were deparaffinized, hydrated, submitted to antigenic unmasking, incubated with appropriate primary anti-claudin-2 (MyBioSource, MB 58203337) and anti-claudin-4 (Rockland, 600401AL4) and secondary antibodies, and developed with specific kits (Dako LSAB2 System-HRP, K0673). The results were evaluated by morphometry using the ImageJ imaging program (NIH).

### Cytokine Determination

ELISA kits (BioLegend) were used to analyze the cytokine production (IL-6, IL-1β, IFN-γ, TNF, IL-23, IL-17, IL-10, and TGF-β) in the colon tissue. The tissue homogenate was prepared in a normal saline solution supplemented with a protease inhibitor. After homogenization and centrifugation, the supernatants were collected to detect the cytokine concentration according to the manufacturer’s protocol.

### Determination of SCFA

The concentration of SCFA was analyzed by HPLC from the homogenate of the cecum prepared with 50 mg of the tissue in 500 μL of acidified deionized water. The SCFA mixture was prepared from solutions of acetic acid, propionate, and butyrate with purity ranging from 99.5 to 99.9%, in varying concentrations in multiples of 5000 μM (25,000 μM to 15.62 μM) to generate the calibration curve of each SCFA. The Shimadzu LC Solution HPLC analytical system was used with ion exclusion column coupling SUPELCOGEL#C-610H (59320-U) for chromatographic separation. Elution was performed with a mobile phase composed of sulfuric acid (H_2_SO_4_) at 0.01 N at a rate of 1 mL^–1^ min. 40 μL of each sample was injected, and elution monitoring was performed at 210 nm, in a total analysis time of 35 min. The concentrations were calculated from the equations generated in the calibration curves and expressed in micromolar units (μM).

### Detection of Secretory IgA

ELISA kits (BioLegend) were used to detect the concentration of SIgA in feces. Fecal homogenate was prepared in a normal saline solution supplemented with a protease inhibitor. After homogenization and centrifugation, supernatants were collected to detect the concentration according to the manufacturer’s protocol.

### TRPV1 Expression

TRPV1 protein expression was made from the colonic tissue incubated with anti-TRPV1 (SC398417, Santa Cruz Biotechnology) overnight at 4 °C, and Alexa Fluor 594 (R37119 Thermo Fisher Scientific) was used to detect the markings. The images were captured in an ApoTome.2 ZEISS microscope, and the fluorescence intensity was analyzed with the image analyzer software Image-J (NIH).

### Statistical Analysis

All results were expressed as mean and standard error and evaluated by one-way or two-way ANOVA, followed by a post-test of multiple comparisons by Tukey’s test for the analysis of more than two groups or Student’s *t*-test for comparison of two groups and by Kruskal–Walis test, followed after the test of multiple comparisons by Dunn’s test for analyses of more than 2 groups, or the Mann–Whitney test for comparison of two groups. Statistical analyses were performed using GraphPad Prism 8.0 (GraphPad Software, San Diego, California – USA). Differences between the means of the groups with p values of ≤0.05 were considered statistically different.

## Supporting Information Available

Composition of the creams (100 g), chromatographic analysis of capsaicin and vehicle (placebo) creams, cream analysis by HPLC, and radiolabeling of capsaicin with ^99m^technetium (PDF)
