# Supplementary material for: Cutaneous Application of Capsaicin Cream Reduces Clinical Signs of Experimental Colitis and Repairs Intestinal Barrier Integrity by Modulating the Gut Microbiota and Tight Junction Proteins
Source: ACS Pharmacol Transl Sci. 2024 Jun 12;7(7):2143–53. doi: 10.1021/acsptsci.4c00207 (PMC11249629; doi:10.1021/acsptsci.4c00207)
Supplement: Supplementary file 1 — pt4c00207_si_001.pdf [file pt4c00207_si_001.pdf]

SUPPLEMENTARY INFORMATION

Cutaneous application of Capsaicin cream reduces clinical signs of experimental colitis and repairs intestinal barrier integrity by modulating the gut microbiota and tight junction proteins.

Elandia A Santos[*1]; Janayne L Silva[1], Paola C.L. Leocádio[1], Maria Emilia R. Andrade [2], Celso M. Queiroz-Junior[3], Nathan S. S. Oliveira[1], Juliana L. Alves[1], Jamil S. Oliveira[1], Edenil C. Aguilar[6], Kennedy Boujour[4,5], Bruno Cogliati[4], Valbert N. Cardoso[2], Simone Odilia A. Fernandes[2], Ana Maria C. Faria[1], Jacqueline I. Alvarez-Leite[1].*

[1]Departamento de Bioquímica e Imunologia – Instituto de Ciências Biológicas - Universidade Federal de Minas Gerais (UFMG)

[2]Departamento de Análises Clínicas e Toxicológicas - Faculdade de Farmácia da UFMG

[3]Departamento de Morfologia – Instituto de Ciências Biológicas - (UFMG)

[4]Departamento de Patologia Animal - Universidade de São Paulo (USP)

[5]Unity of Biochemistry Membrane and Transport, Department of Cellular Biology and Infection, Institut Pasteur, Paris 75724 Paris Cedex 15, France

[6]Icahn School of Medicine at Mount Sinai, USA

* Corresponding author (elandianutri@gmail.com)

Table S1. Composition of the creams (100g)

Composition	Base Cream (Vehicle)	Capsaicin 0.075%
Butil-hidroxitolueno – BHT	0.05	0.05
Capric/caprylic acid triglycerides	0.05	0.05
Sodium EDTA	0.1	0.1
Imidazolidinylurea (50%)	0.6	0.6
Liquid paraffin	3.0	3.0
Silicone	4.5	4.5
Xanthan gum	5.0	5.0
Paraben preservative solution	3.3	3.3
Nonionic self-emulsifying wax	9.0	9.0
Capsaicin*	-	0.075
Distilled water	q.s.p 100g	q.s.p 100g

*Pharmacy Nature Derme (Belo Horizonte-MG)

Supplementary information (S2) Chromatographic analysis of capsaicin cream and placebo

Confirmation of the purity of the creams was made by reversed phase chromatography (HPLC). The samples were prepared as follows: 100 mg of base cream and capsaicin cream were weighed in separate microtubes and homogenized in 1 mL of Merk methyl alcohol (99.9% purity). For the purpose of comparison with the samples, we prepared a 5 mmol capsaicin methanol solution as standard. The HPLC analytical system used was the Shimadzu LC Solution. An ACE C18 column (4.6 x 250 mm, 5 μm) was used for chromatographic separation and maintained at 40°C. Elution was performed with a mobile phase composed of methanol:water (50:50 v/v) at a rate of 1 mL^{-}1min. The 40 L samples μwere injected and the elution was monitored at 280 nm.

Supplementary figures

Figure S1 - Standard capsaicin 5uM chromatograms.

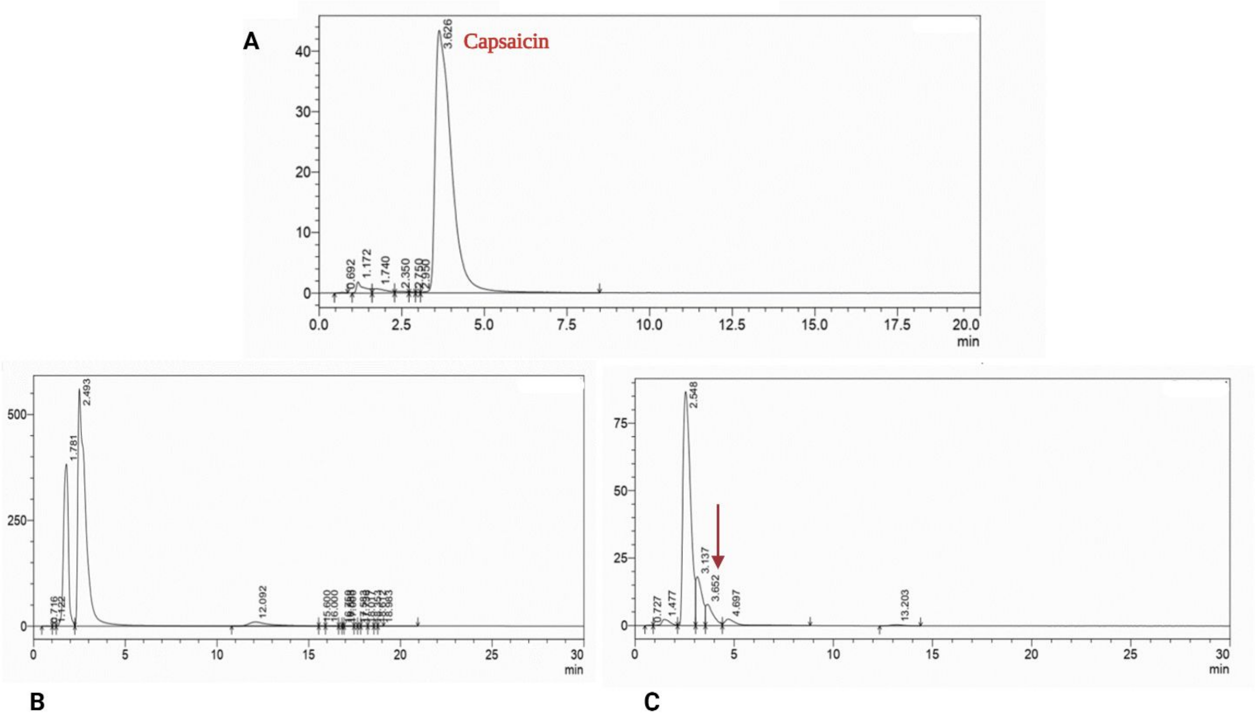

Figure S1: Cream Analysis: (A); Standard capsaicin 5uM (B) base cream (vehicle) 100mg; (C) and capsaicin cream 100mg at 0.075% (C). The arrow indicates the CAP retention time (3.626 min)

Supplementary information (S3) - The radiolabeling of capsaicin with ^{99m} Technetium

The radiolabeling of capsaicin with ^{99m} Technetium (^{99m}Tc) was performed as follows: Previously, several tests were performed in the Radioisotope Laboratory in order to define the best labeling standards. Synthetic capsaicin (N-Vanillylnonanamide – Sigma V9130) and ^{99m}Tc in the form of sodium pentecinate (Na^{99m}Tc^{O4}) obtained from the ^{99}Molybdenum/^{99m}Technetium generator (IPEN/CNEN, São Paulo, Brazil) were used. The radiolabeling of capsaicin and determination of the radiolabeling yield were based on methodology proposed by Hosseinimehr, Ahmadi; Taghvai, 2010 (Hosseinimehr, Ahmadi, ; Taghvai 2010). Initially, capsaicin was solubilized in methanol (1mg^{-}1mL). Then, 200 µL of SnCl2 in 0.01M HCL (1mg^{-}1mL) and 400 µL of phosphate buffer saline (PBS) were added to a microtube of 1000 µL of capsaicin solution, adjusting the pH 6, using an aqueous solution of 0.1 N NaOH. Subsequently, the vial was sealed for vacuum. Next, 0.2 mL of saline containing 148 MBq of Na^{99m}TcO$_4$ was added. The reagent mixture was kept at room temperature for 30 min.

After labeling, the reagent mixture was filtered using a Sep-Pak C-18 filter. The labeled capsaicin was eluted with 5 ml of methanol, which was subsequently evaporated using a heat blower. The in vitro *radiolabeling yield* was determined by thin layer chromatography. Silica gel was used as the stationary phase and saline and methanol were used as mobile phases to determine the percentage of free technetium ($^{99m}TcO_4^-$) and hydrolyzed technetium ($^{99m}TcO_2$), respectively. The radiolabeling yield was determined using the following formula: % ^{99m}Tc-capsaicin = 100 – (%$^{99m}TcO_4^-$ + %$^{99m}TcO_2$).

Scintigraphic images were performed in order to confirm the result of radiolabeling purity obtained by thin layer chromatography and also to verify the stability of the complex in *vivo*. To do

this, the methanol was completely evaporated from the reagent mixture. Subsequently, ^{99m}Tc-capsaicin was reconstituted in 70% ethanol and added to the ointment. After homogenization, 100 mg of the ointment was applied to C57BL/6 mice in the control (n = 3) and DSS (n = 3) groups. At 2 h and 4 h after administration, the animals were anesthetized with ketamine and xylazine solution and then placed in dorso-ventral decubitus under the gamma chamber (NucleineTM TH 22, Hungary). A 20% symmetry window was used for peak energy of 140 keV and a low energy collimator was used to direct the rays. The images were obtained for 5 minutes and stored in a 256 x 256 pixel matrix.
